# TCMGeneDIT: a database for associated traditional Chinese medicine, gene and disease information using text mining

**DOI:** 10.1186/1472-6882-8-58

**Published:** 2008-10-14

**Authors:** Yu-Ching Fang, Hsuan-Cheng Huang, Hsin-Hsi Chen, Hsueh-Fen Juan

**Affiliations:** 1 Institute of Molecular and Cellular Biology, National Taiwan University, Taipei, Taiwan; 2 Institute of Biomedical informatics & Center for Systems and Synthetic Biology, National Yang-Ming University, Taipei, Taiwan; 3 Department of Computer Science and Information Engineering, National Taiwan University, Taipei, Taiwan; 4 Department of Life Science, National Taiwan University, Taipei, Taiwan; 5 Graduate Institute of Biomedical Electronics and Bioinformatics, National Taiwan University, Taipei, Taiwan; 6 Center for Systems Biology and Bioinformatics, National Taiwan University, Taipei, Taiwan

## Abstract

**Background:**

Traditional Chinese Medicine (TCM), a complementary and alternative medical system in Western countries, has been used to treat various diseases over thousands of years in East Asian countries. In recent years, many herbal medicines were found to exhibit a variety of effects through regulating a wide range of gene expressions or protein activities. As available TCM data continue to accumulate rapidly, an urgent need for exploring these resources systematically is imperative, so as to effectively utilize the large volume of literature.

**Methods:**

TCM, gene, disease, biological pathway and protein-protein interaction information were collected from public databases. For association discovery, the TCM names, gene names, disease names, TCM ingredients and effects were used to annotate the literature corpus obtained from PubMed. The concept to mine entity associations was based on hypothesis testing and collocation analysis. The annotated corpus was processed with natural language processing tools and rule-based approaches were applied to the sentences for extracting the relations between TCM effecters and effects.

**Results:**

We developed a database, TCMGeneDIT, to provide association information about TCMs, genes, diseases, TCM effects and TCM ingredients mined from vast amount of biomedical literature. Integrated protein-protein interaction and biological pathways information are also available for exploring the regulations of genes associated with TCM curative effects. In addition, the transitive relationships among genes, TCMs and diseases could be inferred through the shared intermediates. Furthermore, TCMGeneDIT is useful in understanding the possible therapeutic mechanisms of TCMs via gene regulations and deducing synergistic or antagonistic contributions of the prescription components to the overall therapeutic effects. The database is now available at .

**Conclusion:**

TCMGeneDIT is a unique database that offers diverse association information on TCMs. This database integrates TCMs with biomedical studies that would facilitate clinical research and elucidate the possible therapeutic mechanisms of TCMs and gene regulations.

## Background

Traditional Chinese Medicine (TCM), a complementary and alternative medical system in Western countries, has been used to treat various diseases over thousands of years in East Asian countries. The fundamental principles of TCM are based on the Yin-Yang doctrine, the symbolic way of designating opposing forces, and the five element theory that everything in the Universe is dominated and balanced by the five elements, wood, fire, earth, metal and water [1]. The therapeutic mechanism of TCM focuses on enhancing human body's resistance to diseases by improving the inter-connections among self-controlled systems and integrating the human body with the environment [2]. The practice of TCM involves physical therapy such as acupuncture and chemical therapy using materials originating from plants, minerals and animals, while TCM natural products may comprise one or more herbs in the form of decoctions [1,3]. In recent years, many herbal medicines have been reported to be associated with various symptoms or diseases and may exhibit a variety of effects through regulating a wide range of gene expressions or protein activities, such as the anti-tumor effects through the induction of the DR3 and DR4/5 death receptors in human monocytic leukemia cells [4], the anti-inflammatory potential through the inhibition of cytokine, iNOS and COX-2 expression via the NF-kappaB pathway [5], the hepatoprotective effect through the activation of pregnane X receptor in rats [6], the expression of bcl-2 oncogene in gastric precancerous lesions [7], the up-regulation of proapoptotic Bax expression in human bladder carcinoma T24 cells [8], the anti-aging effect on the cerebral gene expression levels in the senescence-accelerated mouse prone 8/Ta [9], and the alteration of gene expressions of hepatic stellate cells against hepatic fibrosis [10]. Therefore, studies about the connections between the TCMs, genes and diseases are emerging and important. In this study, TCM primarily means the natural products developed from plants and animals.

The primary goal of text mining is to retrieve knowledge hidden in text and to present the distilled knowledge such as associations, patterns to users in a concise form [11]. With the rapid increase of available TCM data, there is an urgent need to explore these resources effectively from the large quantity of literature [12]. Over the past few years, several studies have been made on the mining or extraction of TCM information from literatures, such as understanding ZHENG (syndrome) in TCM in the context of neuro-endocrine-immune network [13], extraction of clinical TCM formula data from literature [14], finding functional community of related genes using TCM knowledge, symptom complex [15], and extracting knowledge of drugs and formulae from semi-structured text [16]. Many databases were developed to provide researchers with different aspects of TCM information, but few of them are published, presented in English, and freely accessible. For instance, TCM-ID contains information on prescriptions, herbs, herbal ingredients and 3D structures of partial herbal ingredients [17], the Chinese herbal medicines toxicology database holds herb monographs with a full toxicity profile and grading plus scientific evidences [18], the 3D structure database consists of chemical compounds isolated from Chinese traditional herbs [19], and the TCM database includes basic herb data, chemical components, molecular structures, and bioactive data [20]. However, to date, there has been no attempt to develop a database integrating information about TCMs, genes and diseases using text mining. Furthermore, little attention has been given to build a knowledge base containing associations between TCMs and genes.

The purpose of this paper is to present a database, TCMGeneDIT, providing associations about TCMs, genes, diseases, effects and ingredients, and relations between TCM effects and effecters from vast amount of biomedical literature. Information about protein-protein interactions and biological pathways from public databases is also accessible. We illustrated the value of TCMGeneDIT by its utility in many aspects and two use cases about the possible therapeutic mechanisms of TCMs and synergistic or antagonistic contribution of the prescription component. The integrated genomics, proteomics, and text mining data, and user-friendly web interfaces in TCMGeneDIT make it a one-stop site for TCM and modern biomedical researchers. Thus, the database would facilitate clinical research and offer a better understanding for researchers of the TCM and gene regulation-involved therapeutic mechanisms.

## Methods

### Data sources and contents

**Figure 1:**
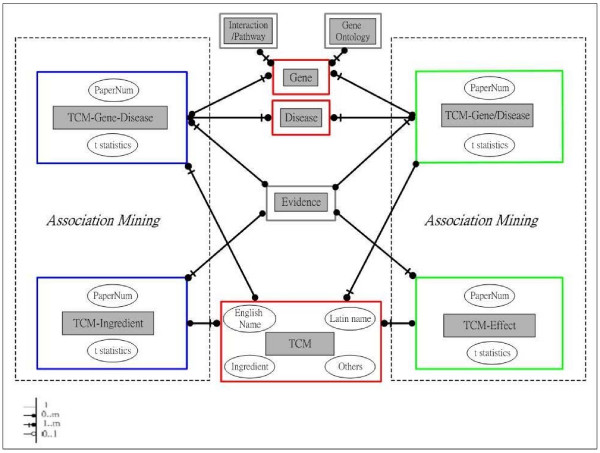
**The simplified relational scheme of TCMGeneDIT**. Each gray box represents an entity with various major attributes characterized by oval-shape. For instance, each TCM herb may be associated with one or many genes involving in several signaling pathways and have many interacting partners. Theses associations may be related to the therapeutic mechanisms for certain diseases and could be supported by scientific evidences.

TCMGeneDIT, a relational database, is implemented by MySQL, Perl and PHP programming languages in the Linux environment. Figure 1 presents the simplified relational scheme of our database. Each TCM herb may contain various ingredients and involve several biological pathways through its interactions with numerous genes, which could be supported by one or many literature evidences; and these interactions may help in the discovery of new therapies for diseases.

**Figure 2:**
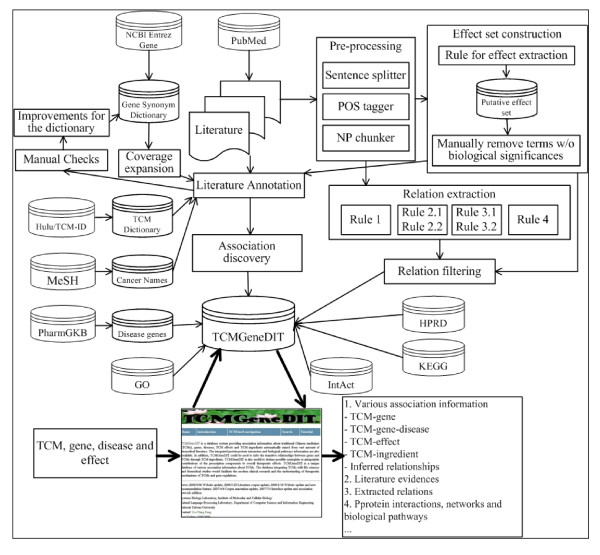
**The text mining approach and information integration for TCMGeneDIT**. Literature corpus about TCMs was collected from PubMed and used for entity annotations and information extraction. Annotated documents were mined based on hypothesis test and collocation analysis to discover entity associations. On the other hand, the raw corpus was pre-processed with public tools and several rules can be applied to the processed sentences for extracting the relations between effecters and effects. The constructed effect set was used to filter candidate relation and literature annotation. Protein-protein interactions and biological pathways were integrated into our database and disease candidate genes from PharmGKB were used for transitive inference. Users can access the database via the web interface. Thick arrow indicates the basic work flow of TCMGeneDIT.

TCM herb data were collected and integrated from the HULU TCM professional web site  and TCM-ID. As of now, our database includes over 2,000 TCM entries. In addition, ingredient data obtained from the Chinese medicine resource web  serve as a supplementary to the above-mentioned two sources. The general human gene information, including official gene symbol, aliases, descriptions and functions were retrieved from NCBI Entrez Gene [21]. The scope of the dictionary on gene names was expanded by ProThesaurus-Wiki [22]. Disease names were extracted from the heading and entry term fields in the disease (C) section of the medical subject headings vocabulary (MeSH), except subsection C22, animal disease and C23, pathological conditions, signs and symptoms. The entry terms could be regarded as the synonyms of the disease names. Nonspecific MeSH terms like 'disease', 'cancer' or 'neoplasm' were excluded. Currently, the database covers 13167 gene and 3360 disease entries, respectively. 38,072 MEDLINE abstracts were collected through PubMed with herbal medicine generic names and specific epithets. The relationships between genes and diseases were collected from PharmGKB [23]. The protein-protein interaction data, including interacting partners, interaction types and detection methods, were obtained from HPRD [24] and IntAct [25]. The biological pathway information describing pathway types, regulations for genes, and/or experiments was retrieved from HPRD, KEGG [26] and CGAP [27]. Most association information and potential TCM effects were mined and extracted from literature abstracts. Association visualization was implemented using Graphviz, open source graph visualization software . Figure 2 shows our text mining approach and information integration for developing TCMGeneDIT.

### Literature corpus annotation

The TCM names, gene names, MeSH disease terms, TCM ingredients and effects were used to annotate the literature corpus. All words were queried against documents in a case-insensitive and exact string matching manner.

We used the generic names and specific epithets such as *Ganoderma lucidum *of the TCM scientific names to match text. In addition, the abbreviated forms, for example, *G. lucidum *and *G lucidum *were considered.

After initial gene name identification, the most recent 100 gene-annotated documents were manually examined to reduce ambiguous gene symbols that may represent genes, diseases, cell lines, experiment techniques or other terms simultaneously. If any ambiguous terms were frequently matched in the text and do not mean genes, these gene symbols would be regarded as stop words and excluded from the gene name dictionary. We then annotated gene names in the literature again with the improved dictionary. In other words, the process can be executed after every update of the literature corpus so that the dictionary could be more specific and precise to the research domain. The approach mainly followed our pervious work [28]. Since term ambiguity may occur between gene symbols (for example, p38 may represent MAPK1 or MAPK14), the NCBI Entrez gene IDs of the official gene symbols was assigned to the symbols or their aliases, if any, for the following association discovery.

In the disease name lexicon, if a term contains comma such as 'Tumors, Breast', all words of this term were individually queried against documents and the word sequence was ignored. If these words can be matched in a document, we assumed the document was identified by the term. Other terms without comma were directly queried against text.

The putative TCM effect list was generated by rule-based approach mentioned below. Synonyms such as 'anti-tumor' and 'anti-tumour' were manually grouped before annotation.

### Association discovery

Annotated literature corpus was then used to find various associations including (TCM, gene), (TCM, disease), (TCM, gene, disease), (TCM, ingredient), (TCM, effect) and (gene, ingredient). The concept to discover associations was based on hypothesis testing and collocation, an expression consisting of two or more words that tend to occur together [29]. The statistical methods based on co-occurrences can be very fast and useful for getting precise information [30]. One can observe the co-occurrence of these entities in abstracts and calculate how improbable it is to observe a certain level of co-occurrences by chance. The null hypothesis (H_0_) is that the co-occurrences between TCM and entities are by chance, and so the probability of the terms appearing concurrently is given by: *P*(term^1^term^2^) = *P*(term^1^)*P*(term^2^). Since a term may include multiple synonyms, the occurrence probability of the term has to be represented by all its synonyms to reflect the real weight of this term. For example, if gene A has three synonyms, gene A1, gene A2 and itself and each of them occurs one, two and three times in text, respectively, then the total occurrence number of gene A is six in fact. The *t *test that looks at the mean and variance of a sample of measurements was then used to discover collocations. The *t *value was calculated with the following formula: $t=\frac{\bar{x}-\mu }{\sqrt{\frac{s^{2}}{N}}}$ where $\bar{x}$ is the observed probability, μ is the expected probability, *s*^2 ^is the sample variance and *N *is the sample size, the total number of tokens in the corpus. If the *t *value is larger than 2.576, 2.326, 1.960 or 1.645, we can reject the null hypothesis with 99.5%, 99%, 97.5% or 95% confidence, respectively. In other words, associations discovered by chance would have low statistical values and not be shown or rank low in the results based on the threshold set by users.

### Rule-based information extraction

To extract the relations between effecters and effects from literature, sentences were part-of-speech (POS)-tagged by Genia Tagger [31] and noun-phrase (NP) chunks were identified by NPchunker [32]. The effecter and effect relations were extracted by matching preprocessed sentences with manually designed four rule patterns for high precision. For example, "*[The/DT anti-androgenic/JJ activity/NN] of/IN [the/DT ethanol/NN extract/NN] of/IN [the/DT fruiting/VBG body/NN] of/IN [Ganoderma/NN lucidum/NN] has/VBZ been/VBN previously/RB reported/VBN*./." is a POS-tagged and NP chunked sentence where each square bracket represents a NP.

Rule for effect extracts terms POS-tagged as adjectives and followed by effect-related keywords (for example, 'anti-tumor activity'). The corresponding pattern is as follows: [? (effect term/JJ) (selected keyword)/NN] where the selected keywords for effect identification include 'effect', 'activity', 'potential', 'property' and 'function', parenthesis represents priority, | denotes 'or' and ? denotes optional argument. The extracted terms without biological significances such as 'important' or 'great' were manually excluded.

Rule 1 extracts effect and effecter NP chunks connected by *of*. The effecter names could be composed of multiple chunks. The corresponding pattern is as follows: [? (effect) (keyword)/NN] of/IN [effecter 1] (((of | from)/IN [effecter 2] of/IN [effecter 3])|nil) where effecters 1, 2 and 3 represent the first, second and third NPs in the effecter name, respectively. For example, the above-mentioned example sentence can be matched by this rule and the effecter, '*the ethanol extract of the fruiting body of Ganoderma lucidum*' and effect, '*anti-androgenic*' are able to be extracted.

Rule 2.1 extracts effecter and effect NP chunks connected by active verbs. The effecter names are composed of two NP chunks connected by *of *or *in*. The following is the corresponding pattern: [effecter 2] (of | in)/IN [effecter 1] (modal/MD|nil) verb/VB [? (effect) (keyword)/NN]. For instance, a sentence matched by this rule can be '*the polysaccharides fraction of G. lucidum exhibited significant anti-tumor effect' *and effecter, '*the polysaccharides fraction of G. lucidum*', and effect, '*anti-tumor*', can be extracted.

Rule 2.2 is similar to Rule 2.1, but extracts one or more effecters connected by *and*. The following is the corresponding pattern: [1^st ^effecter] (((n-2 effecters) and/CC [n^th ^effecter])|nil) (modal/MD|nil) verb/VB [? (effect) (keyword)/NN] where n is greater than or equal to two if there are multiple effecters mentioned. The sentence, for example, '*Lingzhi and San-Miao-San capsules might exert a beneficial immunomodulatory effect*' is able to be matched by this rule and effecters, '*Lingzhi and San-Miao-San capsules*', and effect, '*immunomodulatory*', can be extracted.

Rule 3.1 extracts effecter and effect NP chunks connected by past participle verbs that are frequently observed in English for describing research discoveries. The corresponding pattern is as follows: [effecter 2] (of | in)/IN [effecter 1] (was | were)/VBD keyword/VBN to/TO verb/VB [? (effect) (keyword)/NN] where the keywords for VBN include 'found', 'shown', 'demonstrated', 'known', 'concluded', 'reported', 'proved', 'seen', 'suggested' and 'observed'. For example, this rule can match the sentence, '*hot water extract of the mushroom Ganoderma lucidum was found to exhibit antioxidative effect*' and '*hot water extract of the mushroom Ganoderma lucidum*' and '*antioxidative*' can be extracted as effecter and effect, respectively.

Rule 3.2 is similar to Rule 3.1, but extracts one or more effecters connected by *and*. The corresponding pattern is as follows: [1^st ^effecter] (((n-2 effecters) and/CC [n^th ^effecter])|nil) (was | were)/VBD keyword/VBN to/TO verb/VB [? (effect) (keyword)/NN]. For instance, effecter, '*APBP*', and effect, '*antiviral*', are able to be extracted from the sentence, '*APBP was shown to have potent antiviral activity*'.

Rule 4 extracts effecter and effect NP chunks connected by appositives modifying effecters, and verb phrases. The following is the corresponding pattern: [effecter],/, appositive,/, (modal/MD|nil) verb-phrase [(effect) (keyword)/NN]. For example, the sentence, '*LZ-8, a new and recently discovered immunomodulator from Ganoderma lucidum, has been shown to have immunosuppressive activity*' can be matched and effecter, '*LZ-8*', and effect, '*immunosuppressive*', are able to be extracted, respectively.

The candidate relations having following conditions were excluded: (i) the extracted effects can't be identified in the effect set; (ii) the effecters are non-specific terms such as compound and sample; (iii) the start words of the effect NP chunks are 'no'.

### Transitive association

We inferred transitive associations about TCMs according to Swanson's ABC model where if A and B are related, and B and C are related, then A and C might be indirectly related [33]. For TCM and gene association inference, we assumed that gene (A) activities may be regulated by various ingredients (B) isolated from the TCMs (C) and estimated the generated relationships by the formula: $\sum_{i=1}^{n}t_{AB_{i}}\cdot t_{B_{i}C}$, where n is the number of ingredients involved, $t_{AB_{i}}$ denotes the *t *value for gene (A) and ingredient (B_i_) and $t_{B_{i}C}$ denotes the *t *value for ingredient (B_i_) and TCM (C). As the work we referred, if the relationships between A and B, and B and C are more significant (higher *t*_AB _and *t*_BC_), the inferred associations between A and C could be more reliable (higher score calculated). Similar concept could be applied to correlate TCMs (A) with diseases (C) via known disease candidate genes (B). If the relationships between TCMs and these genes are more significant, the associations between TCMs and diseases contributed by these genes could be more promising.

## Results

### Utility

**Figure 3:**
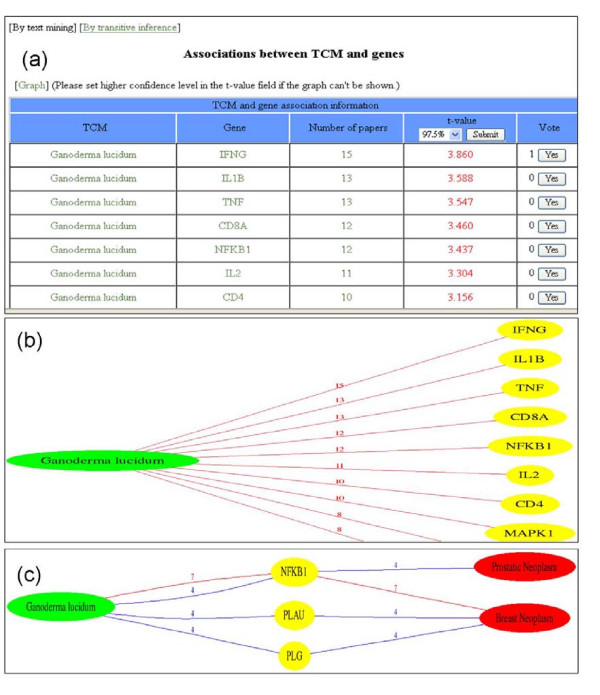
**The TCM and gene associations and visual information representations**. (a) The *Ganoderma lucidum *and gene associations from text mining. Detailed information about TCM and genes could be accessed by following the links themselves. Literature evidences supporting the associations are available through following the links indicating the number of paper. Confidence thresholds are able to be selected. Users with domain knowledge can recommend the associations they think are correct. Moreover, TCM and gene associations could be discovered by transitive inference. (b) TCM and gene association graph. TCM and genes are represented by green and yellow nodes, respectively. The edges between them are colored according to *t *values (please see the text). The numbers on the edges mean how many literatures may support the associations. (c) TCM, gene and disease association graph. Red nodes mean diseases.

The database can be accessed by TCM Latin names, gene symbols, gene-related keywords, disease names and TCM effects to find various associations and integrated information. TCM, gene and disease associations could be presented in network graphs. In TCMGeneDIT, TCM tree browser is also available for users unfamiliar with TCM Latin names. The main search interface is composed of three sections including (i) TCMs and Genes, (ii) Diseases, (iii) TCM effects, and different options. Figure 3 shows one of the major features of TCMGeneDIT, the associations between TCM and genes, and visual representations for (TCM, gene) and (TCM, gene, disease).

In order to retrieve association information regarding *Ganoderma lucidum*, for example, users can select 'TCM Herb' search type, input *Ganoderma lucidum *as the search term and then click on the "Search" button. If the search is matched, the TCM information, including three links for TCM-Gene, TCM-Disease and TCM-Gene-Disease, ingredients collected from other databases and mined from literature, potential effects from text mining, and putative relations between effecters and effects extracted from the text will be shown. All association information is presented with *t *values in different colors, red (>2.576), green (>2.326) and blue (>1.960). Users can then follow the links for evidences in sentences from the literature, highlighted keywords, identified genes, journals and publication years. To examine the relationships between *Ganoderma lucidum *and genes, users can click on the TCM-Genes link and the returned web page will contain associations ranked by the *t *values and vote number. The vote features were presented for users with domain knowledge to recommend correct associations. Users are able to examine associations with preferred confidence levels. The default value is 97.5%. Evidence extracted from the literature can be accessed by following the link showing the number of paper. Users can then follow the links of gene names for general information and associations between specific genes and different TCMs. Furthermore, users are able to find which genes and diseases may be related to *Ganoderma lucidum *via the link TCM-Gene-Disease. The default results are associations purely generated from collocation analysis. The links of diseases are for users to examine disease descriptions from MeSH and which TCMs may be related to the disease. Additional TCM, gene and disease association information could be accessed by transitive inference, as the relationships between genes and diseases collected from PharmGKB are integrated to the text mining results of TCMs and genes.

To search for association information using gene names, users can select 'Gene Symbol' search type, for example, input 'TNF-alpha' as search term and select the gene of interest in the returned page. If users follow the link of TNF gene ID, a variety of gene information are shown, including gene description, function, two links for TCM-Gene and TCM-Gene-Disease, Gene Ontology annotation, involved biological pathways and interacting protein partners. For those who would like to find the associations of specific disease, such as Alzheimer's disease, with TCMs or both the TCMs and genes, can simply select the disease name in the main search interface. Disease description and three links for TCM-Disease, TCM-Gene-Disease and TCM-Gene-Disease (transitive) will be presented. The transitive disease, TCM and gene associations are inferred via common TCMs and preferred confidence levels for disease-TCM and TCM-gene associations can also be set. The associations between TCMs and particular effects can be accessed in the third section of the main search interface. For example, *Ganoderma lucidum *may be most associated with 'antitumor' effect.

### Inference of the relationships between TCMs and genes through TCM ingredients

In addition to providing associations between TCMs and genes by their co-occurrences, TCMGeneDIT could infer their transitive relationships through the shared intermediates, ingredients. For example, in the initial result page of *Ganoderma lucidum *and gene associations, users can follow the link 'by transitive inference' and select one or more ingredients of interests to perform relationship inference. Then, our system would try to find putative associations between TCMs and genes according to the sorted scores calculated by the above-mentioned formula: $\sum_{i=1}^{n}t_{AB_{i}}\cdot t_{B_{i}C}$. In this example, TNF (Tumor Necrosis Factor) is suggested to be the gene highly associated with *Ganoderma lucidum *if users select polysaccharide and triterpene, the main ingredients of the TCM, to infer the relationships. Potential transitive associations with high scores but not explicitly found in the literature may be regarded as candidates for hypotheses generation.

## Discussion

To evaluate the association information discovered by the collocation analysis, we randomly selected 50 TCMs related to genes and/or diseases, and used precision measurement to estimate the performances. The mined results manually confirmed as correctness were considered true positives. Precision is defined as follows: $\text{precision}=\frac{TP}{TP+FP}$, where TP and FP are the numbers of true positives and false positives, respectively. Table 1 shows the evaluation results for various associations. The precisions of 666 (TCM, gene), 642 (TCM, disease), and 131 (TCM, gene, disease) associations with 95% confidence level are 92.8%, 86.0% and 87.0%, respectively. In addition, the (TCM, effect) associations and TCM effect relations extracted from literature were also evaluated. There are 570 (TCM, effect) associations at 97.5% confidence level with a precision of 96.5%. To evaluate the precision of the extracted relations between effecter and effect, 20 TCM entries were randomly selected in the test set and the precision of the 1185 relations is 91.2%. The analysis of errors in association discovery showed that false positives were primarily caused by ambiguous gene symbols that could not be excluded in the annotation phase (for example, AP-1 may represent JUN gene or *Angelica sinensis *polysaccharide 1) and general MeSH disease entry term such as 'Toxicity, Drug' or 'Poisoning'. The analysis of errors in relation extraction indicated that the most principal sources of errors came from incomplete, incorrect or non-specific enough effecter NPs.

**Table 1:** Evaluation of the associations between TCMs and various entities from collocation analysis

	(TCM, Gene)	(TCM, Disease)	(TCM, Gene, Disease)	(TCM, Effect)
Precision (%)	92.8	86.0	87.0	96.5
Number of associations	666	642	131	570
Minimum confidence (%)	95	95	95	97.5
Number of TCMs contributed to the associations	48	47	23	44

TCMGeneDIT could be used to understand the possible therapeutic mechanisms of TCMs via gene regulations. Take *Ganoderma lucidum *and anti-tumor for example. From the database TCM search, the mining results indicated *Ganoderma lucidum *may be highly associated with anti-tumor effect (t-value>6.1). In addition, the TCM and gene association information showed IFNG (interferon, gamma), IL1B (interleukin 1, beta), TNF, NFKB1 (nuclear factor of kappa light polypeptide gene enhancer in B-cells 1) and IL2 (interleukin 2) genes were significantly related to *Ganoderma lucidum *(t-values >3.3). Furthermore, the functional classifications of Gene Ontology available in the gene search results revealed that most these genes are involved in apoptosis (80%) and cell proliferation (60%), in which both are correlated with tumorigenesis. The findings suggested *Ganoderma lucidum *may have therapeutic effects on cancers through the regulations of apoptosis and cell proliferation. Recent studies have shown that *Ganoderma lucidum *can induce apoptosis of prostate cancer cells (PC-3) via decreasing the expression of NFKB1-regulated genes [34] and *Ganoderma lucidum *is able to inhibit TNF-induced proliferation of human breast cancer cells through modulation of the NFKB1 signaling [35]. On the other hand, several active ingredients such as polysaccharides, triterpenes, ganoderic acids highly associated with *Ganoderma lucidum *(t-values>5) may contribute to the putative mechanisms. As our expected, *Ganoderma lucidum *polysaccharides have been shown that can modulate the concentrations of IL2, IFNG, TNF, and NFKB1 in patients with advanced colorectal cancer [36], and ganoderic acid Me can inhibit tumor growth and lung metastasis through increasing the expressions of IL2, IFNG and NFKB1 [37]. These results indicated that TCMGeneDIT could suggest the possible therapeutic mechanisms involved by TCMs, genes and TCM ingredients, and provide a one-stop site for TCM and modern biomedical researchers.

TCMGeneDIT may also be useful to determine which components of the prescription composite formulae will produce a synergistic effect or an antagonistic action. We applied TCMGeneDIT to 3 immune-related prescriptions, Dang Guei Bu Syue Tang composed of *Astragalus membranaceus *(Huang Ci) and *Angelica sinensis *(Dang Guei), Sheng Mai San composed of *Panax ginseng *(Ren Shen Ye), *Ophiopogon japonicus *(Cun Dong) and *Schisandra chinensis *(Bei Wu Wei), and Sih Jyun Zih Tang composed of *Codonopsis pilosula *(Dang Shen), *Atractylodes macrocephala *(Bai Jhu), *Poria cocos *(Bai Fu Ling) and *Radix Glycyrrhizae *(Wu Jhu Yu). From the database TCM effect search, we could find all herbs to be immune-related such as immunopotentiating, immunological, immunomodulatory or anti-inflammatory. In the case of Dang Guei Bu Syue Tang, both herbs had high t-values, 3.303 and 2.221, respectively, for antitumor effect and were both significantly associated with IL-2 and TNF. Both IL-2, a secreted cytokine important for the proliferation of T and B lymphocytes, and TNF, a multifunctional proinflammatory cytokine secreted by macrophages, are also known to be involved in cytokine-cytokine receptor interaction, which can be found from our database gene search. Thus, *Astragalus membranaceus *and *Angelica sinensis *may synergistically promote the immune effect of Dang Guei Bu Syue Tang. On the other hand, in Sheng Mai San, *Panax ginseng *is highly related to immune or immunomodulatory effects but *Ophiopogon japonicus *and *Schisandra chinensis *are only slightly immune-related (confidence < 95%). Both *Panax ginseng *and *Ophiopogon japonicus *are associated with TNF, while *Panax ginseng *and *Schisandra chinensis *are both related to IL-6 (interleukin 6), IL-4 (interleukin 4) and IL-10 (interleukin 10). In addition to cytokine-cytokine receptor interaction, IL-6, IL-4 and IL-10 are all involved in the regulation of the Jak-STAT signaling pathway and cytokines and inflammatory responses, which are supported by the KEGG and CGAP -integrated gene search results. Therefore, *Panax ginseng *may very possibly be the major component of Sheng Mai San, in which the immune function is produced by synergistic herb pairs. Although more investigation is required to find the underlying therapeutic mechanisms of the various prescriptions, TCMGeneDIT would certainly facilitate the understanding of the roles played by herb components in producing prescription effects.

### Comparison between TCMGeneDIT and TCM-ID

The TCMGeneDIT differs from the TCM-ID in several ways. First, TCMGeneDIT provides association information about TCMs mined and extracted from a large amount of literature based on collocation analysis, as it joins TCMs with biomedical studies and modern life sciences, especially genomics and proteomics. In addition, the transitive relationships between TCMs and genes and/or diseases could be inferred through TCM ingredients and information integration. Furthermore, integrated protein-protein interaction and biological pathway could help to suggest the underlying therapeutic mechanisms of TCMs and genes.

### Future developments

Future research includes expanding database contents by adding information on prescription composite formulae, TCM ingredient structures, as well as integrating the relationships among Western drugs, genes and diseases, and creating TCM Ontology to describe TCMs and their properties and attributes. We are also planning on developing new rules and extend existing patterns for extracting more relations about TCMs. Furthermore, we intend to offer query functions for network visualization, thus enhancing the study of therapeutic pathways involving TCMs and genes.

## Conclusion

We developed a unique database, TCMGeneDIT, which provides association information about TCMs, genes and diseases using scientific text mining, integrating TCMs with biomedical studies. It will not only help facilitate the understanding of therapeutic mechanisms involving TCM and gene interactions, but also be constructive to modern clinical research.

## Competing interests

The authors declare that they have no competing interests.

## Authors' contributions

YCF carried out the database development, design, programming, data collection, text mining analysis, web interface and drafted the manuscript. HCH and HHC helped with the text mining analysis. HCH, HHC and JHF provided constructive suggestions for improving the website and helped to draft the manuscript. JHF initiated the idea for TCMGeneDIT database development. All authors read and approved the final manuscript.

## Pre-publication history

The pre-publication history for this paper can be accessed here:


